# Spatial distribution of oncocerid cephalopods on a Middle Devonian bedding plane suggests semelparous life cycle

**DOI:** 10.1038/s41598-020-59507-0

**Published:** 2020-02-18

**Authors:** Alexander Pohle, Dirk Fuchs, Dieter Korn, Christian Klug

**Affiliations:** 1 Paläontologisches Institut und Museum, Universität Zürich, Karl-Schmid-Strasse 4, 8006 Zürich, Switzerland; 2 SNSB-Bayerische Staatssammlung für Paläontologie und Geologie, Richard-Wagner-Straße 10, 80333 München, Germany; 3 Museum für Naturkunde, Leibniz-Institut für Evolutions- und Biodiversitätsforschung, Invalidenstraße 43, 10115 Berlin, Germany

**Keywords:** Palaeontology, Palaeontology, Statistical methods, Evolution

## Abstract

Reproductive strategies of extinct organisms can only be recognised indirectly and hence, they are exceedingly rarely reported and tend to be speculative. Here, we present a mass-occurrence with common preservation of pairs of late Givetian (Middle Devonian) oncocerid cephalopods from Hamar Laghdad in the Tafilalt (eastern Anti-Atlas, Morocco). We analysed their spatial occurrences with spatial point pattern analysis techniques and Monte Carlo simulations; our results shows that the pairwise clustering is significant, while ammonoids on the same bedding plane reveal a more random distribution. It is possible that processes such as catastrophic mass mortality or post-mortem transport could have produced the pattern. However, we suggest that it is more likely that the oncocerids were semelparous and died shortly after mating. These findings shed new light on the variation and evolution of reproductive strategies in fossil cephalopods and emphasise that they cannot be based on comparisons with extant taxa without question.

## Introduction

Among the two major clades of living cephalopods, the Coleoidea and Nautiloidea, there is a broad range of reproductive strategies. Coleoids were long considered to be invariably semelparous (reproducing only once), while *Nautilus* is iteroparous (giving rise to offspring multiple times)^1^. However, in recent years, squid, octopus and cuttlefish were shown to exhibit a wide range of reproductive strategies and life cycles^2^. Nevertheless, although its reproductive biology is still poorly known, *Nautilus* is unique among living cephalopods in its polycyclic spawning and long life span, in contrast to the monocyclic spawning (which can occur in separate batches or during an extended time period) of the relatively short-lived coleoids^1,2^.

Studies of the reproduction strategies of the mostly externally shelled fossil cephalopods are very rare, largely because the scarcity of soft part preservation strongly limits the possibilities of these investigations. Yet, cephalopods such as ammonoids, bactritids and orthocerids possess small protoconchs and thus eggs and hatchlings; accordingly, they are often considered to be close to coleoids in their reproductive strategy, while cephalopods with large embryonic shells are generally considered to be closer to the living *Nautilus*^3–6^. In addition, nautiloids are usually thought to be K-strategists (type I survivorship), while coleoids, ammonoids, bactritids and partly orthocerids are seen as r-strategists (type III survivorship^3,7^). However, since both K- and r-strategies occur among recent coleoids combined with a semelparous life cycle^2^, these assumptions may be too simple. In addition, the paradigm of K- and r-strategy^8^ has been abandoned in the field of life-history evolution for some time^9^.

Previous studies have focused on life history traits that can be assessed with some reliability. For example, the mode of life of hatchlings can be inferred from the size of the embryonic shell and facies distribution, while the relative size of the hatchling to the adult can be informative about fecundity^5,10^. Iteroparity or semelparity is more difficult to identify in fossil cephalopods as direct evidence is lacking. In some cases, it has been inferred from combinations of the aforementioned life history traits^11,12^; however, these may not always be good predictors. Apparent mass spawning events in the Late Devonian ammonoid *Prolobites* were seen as support for their similarity to coleoids in terms of reproduction^13^. Further mass occurrences of several ammonoid species in the Middle Carboniferous of Arkansas were similarly interpreted as support for semelparity^14^. In another case, ovoviviparity, which also occurs in recent coleoids, has been suggested for the Early Cretaceous ammonoid *Sinzovia sazonovae*^15^. Furthermore, one recent study showed that large Late Cretaceous ammonoids such as *Parapuzosia seppenradensis* had very high fecundity and that they might represent a case of extreme r-strategists^16^. In general, although reproductive strategies are not well known in ammonoids, it appears clear that a wide range of strategies were probably present due to large differences in life history traits and distribution patterns^17,18^. The repeated occurrence of megastriae confined to the adult body chamber of some specimens of the Middle Ordovician tarphycerid *Tragoceras falcatum* is possibly linked with repeated reproduction cycles and thus iteroparity^19^; but again, it is currently impossible to say how widespread this strategy was among its relatives. Apart from these few reports, it is difficult to infer the reproductive strategy of many Palaeozoic cephalopods. Differences in life habits^20^, egg size^21^, sexual dimorphism^22^ and mature modifications^23^ suggest that at least a similar range of different reproductive strategies existed in extant and extinct cephalopods.

While ammonoids are comparatively well-studied in general, many of the other externally shelled cephalopods – traditionally classified within the Nautiloidea, although alternative classification schemes exist^24^ – need revision and further studies. Especially palaeobiological aspects have received relatively little attention in the past. Members of the order Oncocerida occur in Ordovician to Carboniferous strata. They are characterised by diverse conch morphologies, although most taxa are breviconic, i.e. having a short, straight or slightly curved conch that is rapidly expanding^25^. In terms of reproduction of oncocerids, sexual dimorphism was suggested for a number of species^22^ and mass occurrences of adult specimens in shallow water deposits were interpreted as indication for mass spawning events^26^. In addition, it was proposed that oncocerids and some related taxa might have been ovoviviparous, but conclusive evidence is lacking^26^. Remarkably, oncocerids often display extreme mature modifications in the form of more or less strongly contracted or constricted apertures^25^. It is unclear, whether these modifications primarily had a protective^23^, buoyant^27^ or reproductive function^26^. In any case, the modifications show that the animal underwent significant ontogenetic changes upon reaching adulthood. These ontogenetic trajectories have been interpreted as resulting from a change in mode of life from benthic to nektobenthic^6^, but whether breviconic oncocerids were truly nektobenthic has recently been called into question^28^.

Like many other breviconic nautiloids, remains of oncocerids are not overly common in the fossil record^29^. In that regard, the late Givetian (Middle Devonian) “lower marker bed” at Hamar Laghdad (Tafilalt, Morocco) is one of the few examples, where they occur in large numbers^30,31^. Interestingly, breviconic oncocerids of the genus *Acleistoceras* were reported to be abundant in the late Givetian Lower Cedar Valley Group of Iowa^32^. Similarly, Zhuravleva^33^ described many brevicones assigned to several species of *Ukhtoceras* mainly from the *Pharciceras* ammonoid zone of the Timan Ridge (northwestern Russia) in her comprehensive monograph on Devonian discosorids. According to Dzik^34^, these species are likely synonymous and the genus *Ukhtoceras* belongs to the Oncocerida. At present, we do not know whether these accumulations are of the exact same age or contain the same (or closely related) species. Nevertheless, their occurrence in at least approximately contemporaneous strata is conspicuous and it might suggest that oncocerids experienced a short global blooming phase in the late Givetian.

**Figure 1:**
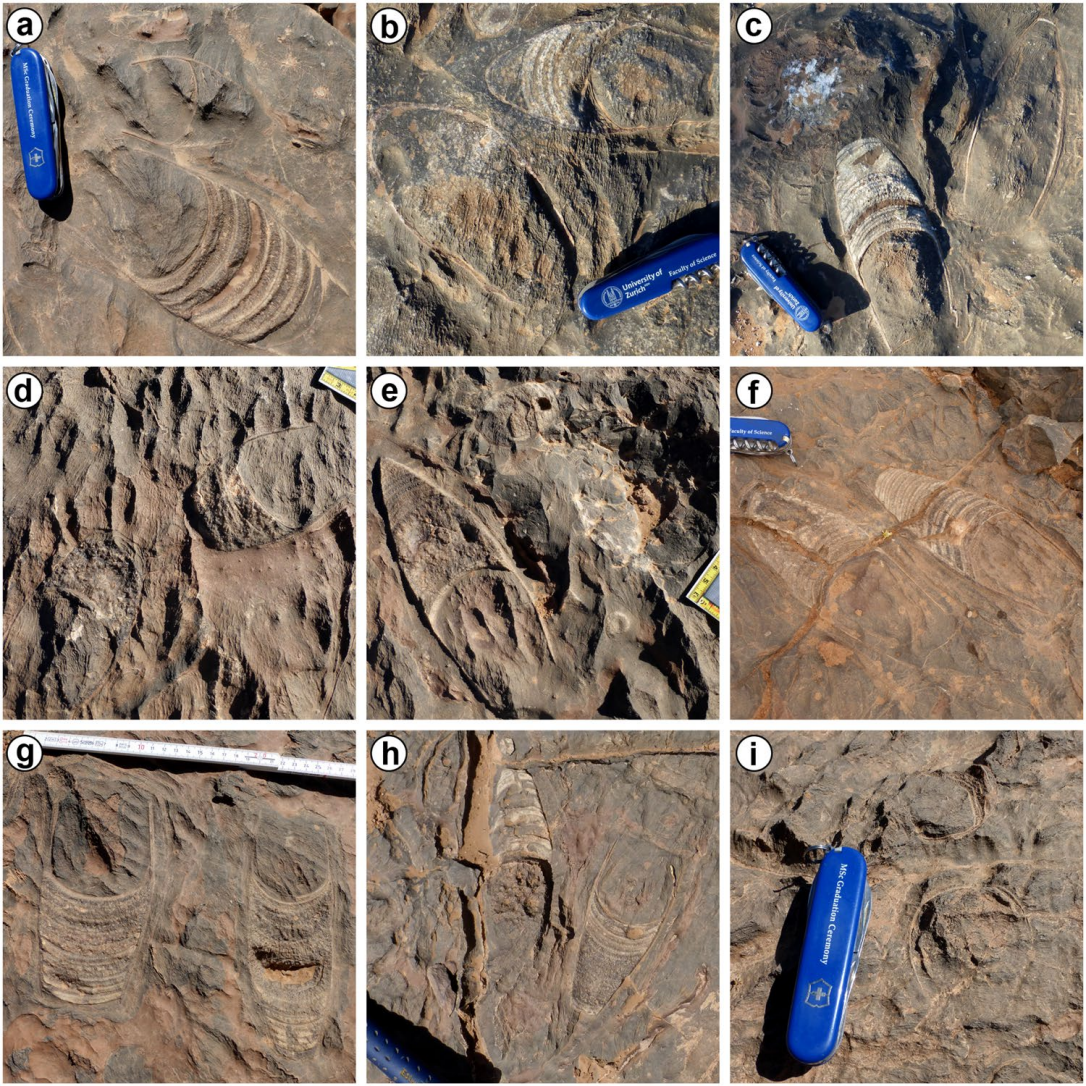
Examples of oncocerid pairs in the “lower marker bed” at Hamar Laghdad. (**a–i**) Most or all of the specimens probably belong to *Pachtoceras*? *laghdadense* Pohle & Klug, 2018. Note that differences in size and shape also depend on the surface of erosion.

Here, we address the question if the oncocerids found at Hamar Laghdad had a semelparous life cycle because of their pairwise distribution on the bedding plane. As stated above, semelparity or iteroparity has previously been hypothesised for a number of fossil cephalopods, but usually this was inferred from a combination of life history traits. Although not ultimately conclusive, the method presented here allows for a more direct test for semelparity in fossil cephalopods and other organisms. Although we analyse only a small part of the exposed bedding plane, closely associated pairs of oncocerids can be found in the whole “lower marker bed” over a great surface area (Fig. 1). We compare the distribution pattern to those of ammonoids and orthocerids on the same bedding plane and discuss alternative hypotheses of taphonomic effects that could have caused or altered the spatial distribution of the oncocerids.

### Geological setting

**Figure 2:**
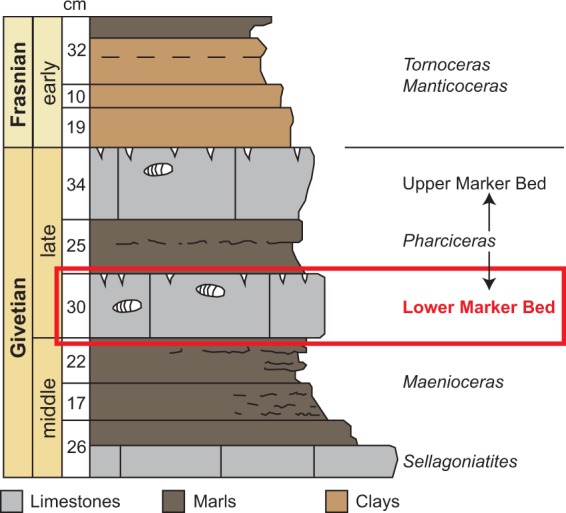
Stratigraphic position of the “lower marker bed”. Adapted from Becker *et al*.^30^.

The Tafilalt in the eastern Anti-Atlas of Morocco has been the subject of a large number of palaeontological studies for almost a century (see^35,36^ for detailed recent literature reviews). The Hamar Laghdad ridge is situated 20 kilometres to the southeast of the town of Erfoud and is famous for its mudmounds that contain a rich and diverse fauna in the late Emsian, but also in the under- and overlying strata from the late Silurian until the late Famennian^30,37^. Nautiloid cephalopods (mostly orthocerids) are common throughout the Devonian succession at Hamar Laghdad and have been mentioned or described on multiple occasions^31,38^. As elsewhere in the Tafilalt, oncocerids also occur at Hamar Laghdad but remain relatively poorly studied; taxonomic studies are almost exclusively confined to the last decade^28,31,39^. Breviconic oncocerids are particularly common in the late Givetian “lower marker bed” at Hamar Laghdad (Fig. 2). In a previous study, we described *Brevicoceras magnum* and *Pachtoceras*? *laghdadense*, but had only few specimens available because they are difficult to extract from the sediments^31^. Judging by the size of many of the specimens studied here, it is possible that many of them belong to *P*.? *laghdadense*. Their regular pairwise clustering might also suggest that at least the pairs belong to the same species, although it is also conceivable that multiple (possibly closely related) species shared the same behaviour.

The “lower marker bed” is characterized by thick limestones that contain abundant trace fossils, pharciceratid ammonoids, the aforementioned oncocerids and moderate numbers of orthocerids^40^. The horizon is widespread in the Tafilalt^41^ and was studied in detail by Ebert^42^, who described it under the name “Unterer *Pharciceras*-Horizont”. The base of this horizon marks the beginning of the upper *hermanni-cristatus* conodont zone and the top is situated in the earliest *disparilis* zone^41,42^.

## Results

**Figure 3:**
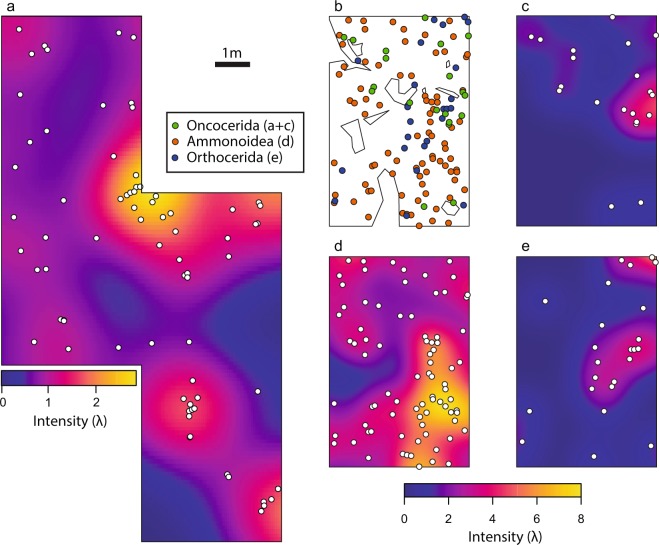
Map of M1 (**a**) and M2 (**b–e**) showing the distribution of cephalopods on the bedding plane and their estimated intensity. An intensity of 1.0 corresponds to one specimen per square meter. Size of the enclosing rectangles: M1 = 8 × 15 m, M2 = 4 × 6 m.

During a first fieldtrip, we documented the spatial positions of 73 oncocerids on one bedding plane with an area of 80 m^2^ (Fig. 3a). We refer to this observation window as M1 in the following text. In a second fieldtrip, we logged the positions of 20 oncocerids, 24 orthocerids and 82 ammonoids on another 24 m^2^ of the same bedding plane, but at another position nearby (M2; Fig. 3b–e). In M2, we also recorded patches where the measuring of specimens was not possible due to poor outcrop conditions due to weathering, holes in the bedding plain, thick sand or dirt or vegetation cover. The area of M2 (excluding these gaps) amounted to 21.8 m^2^ or 91% of the total area of M2. We term the gap-corrected M2 observation window M2x.

### Spatial distribution

**Figure 4:**
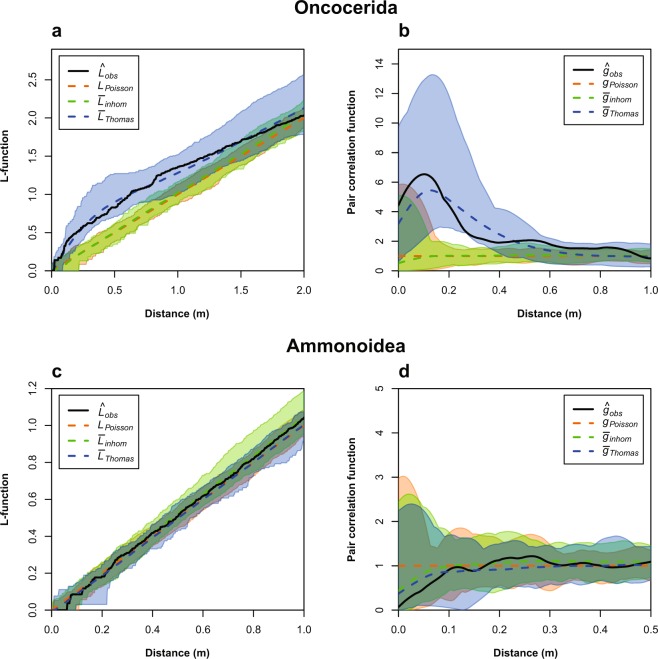
Empirical L- (**a,c**) and PCF-functions (**b,d**) with Monte Carlo simulation envelopes (n = 99) of the M1 oncocerid (**a**,**b**) and the M2 ammonoid (**c**,**d**) pattern. The black lines represent the empirical summary functions (observed data), while the dashed line represent the theoretical mean value of the homogenous Poisson model or the mean value of the Monte Carlo simulations in the case of the inhomogenous Poisson model and the Thomas cluster model. In any case, if the value of function at a given distance r is above the dashed line, this indicates that the points are closer together than expected under the model in question (aggregation of points). If the function lies below the dashed line, this would mean that points would be further apart than expected (segregation). The envelopes provide the minimum and maximum limits for the models and show the expected range of the functions according to different models. The oncocerid pattern fits best to a cluster process, while the ammonoid pattern is best modelled by an inhomogeneous Poisson model.

**Figure 5:**
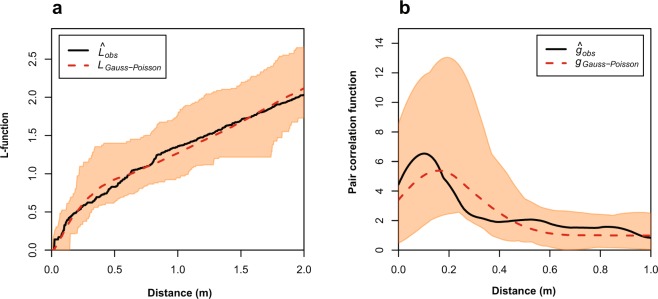
Monte Carlo simulation envelopes of the Gauss-Poisson model of the M1 oncocerid pattern. L-function (**a**) and pair correlation function (**b**). See Fig. 4 for detailed explanations.

We analysed the recorded positions of the fossils by spatial point pattern analysis (see methods section). An overview of the results is shown in Table 1. The mean intensity of the M1 pattern was $\bar{\lambda }$ = 0.91, which corresponds to slightly less than one specimen per square metre on average. The intensity was not homogenous over the whole observation window (Fig. 3a). The empirical L-function and pair correlation function for the M1 oncocerids suggest that the pattern deviates from complete spatial randomness (CSR). The simulation envelopes of Monte Carlo simulations of a homogenous Poisson model with the observed intensity as only parameter (Fig. 4a,b) confirm this observation. The Diggle-Cressie-Loosmore-Ford (DCLF) test resulted in p = 0.01, thus rejecting the null hypothesis of CSR. An inhomogenous Poisson model fitted by the Cartesian coordinates of the observation window yielded very similar results (DCLF test: p = 0.01) and we thus reject this model as well. The fitted parameter values of a Thomas cluster model were as follows: parent intensity κ = 0.5, mean cluster size σ = 1.8 and cluster radius r = 0.19 m. In other words, according to the best-fit cluster model, parent points are randomly distributed with an average of 0.5 points per square meter and subsequently replaced by random clusters that contain a Poisson-distributed number of points with a mean of 1.8 within a radius of 0.19 m. The DCLF test for the Thomas cluster model resulted in p = 0.76, thus the null hypothesis cannot be rejected. The simulation envelopes are also much more similar to the empirical summary functions in comparison with the Poisson models. The fitting of a modified Gauss-Poisson model (Fig. 5) resulted in a parent intensity of κ = 28, a cluster radius of r = 0.28 m and a point retention probability of P = 0.75. In comparison with the Thomas cluster model, parent points are replaced by exactly two random points with a probability of 0.75 and otherwise replaced by one random point. The DCLF test yielded p = 0.95. As both cluster models show non-significant p-values and the simulation envelopes are very similar, it is not possible to choose between the models. Both point pattern processes could lead to a pattern such as on M1.

**Table 1:** Summary of the data and results. Only the M1 oncocerid pattern rejects an inhomogeneous Poisson model. The inferred parameters for the Thomas Cluster model of the other patterns are mainly shown for illustrative purposes, as they are already well described by an inhomogeneous Poisson model. Abbreviations: n = number of recorded specimens, $\bar{\lambda }$ = average intensity, p_hp_ = p-value of the DCLF-test for a homogenous Poisson model, p_ip_ = p-value of the DCLF-test for an inhomogeneous Poisson model using Cartesian Coordinates as density function, p_tc_ = p-value of the DCLF-test for a Thomas Cluster model, κ_**tc**_ = fitted parent intensity for the Thomas Cluster model, σ_**tc**_ = fitted mean cluster radius for the Thomas Cluster model, μ_**tc**_ = fitted mean number of specimens per cluster.

Window	Area	Taxon	n	$\bar{{\boldsymbol{\lambda }}}$	κ_tc_	σ_tc_	μ_tc_	p_hp_	p_ip_	p_tc_
M1	80 m^2^	Oncocerida	73	0.91	0.49	0.17	1.84	**0.01**	**0.01**	0.85
M2	24 m^2^	Ammonoidea	82	3.42	0.04	4.25	76.7	0.11	0.71	0.21
M2x	21.8 m^2^	Ammonoidea	82	3.77	0.21	0.91	18.3	0.22	0.97	0.31
M2	24 m^2^	Oncocerida	20	0.83	0.99	0.19	0.84	0.11	0.52	0.80
M2x	21.8 m^2^	Oncocerida	20	0.92	1.05	0.20	0.88	0.11	0.51	0.76
M2	24 m^2^	Orthocerida	24	1.00	0.91	0.25	1.10	**0.04**	0.58	0.77
M2x	21.8 m^2^	Orthocerida	24	1.10	1.18	0.24	0.94	0.09	0.67	0.84
M2	24 m^2^	All taxa	126	5.25	0.03	3.86	154	**0.01**	0.41	**0.03**
M2x	21.8 m^2^	All taxa	126	5.8	0.01	9.09	462	**0.01**	0.49	**0.03**

On M2, the density of onocerids was similar as in M1 with $\bar{\lambda }$ = 0.83 and $\bar{\lambda }$ = 0.92 for M2x. Orthocerids were slightly more common than oncocerids with $\bar{\lambda }$ = 1.0 on M2 and $\bar{\lambda }$ = 1.1 on M2x, while ammonoids were the most abundant with $\bar{\lambda }$ = 3.42 on M2 and $\bar{\lambda }$ = 3.77 on M2x. Since the number of oncocerids and orthocerids on M2 was very low, the analyses have an only limited statistical power. Nevertheless, visual inspection of the point patterns and summary functions provided valuable insights. The summary functions of the M2 ammonoids deviated from those of the M2 oncocerids and orthocerids. In addition, the M2 oncocerid pattern was close to the M1 oncocerid pattern. The M2 orthocerid summary functions resembled those of both oncocerid patterns; however, pairs were not as apparent in the point pattern. For the oncocerids and orthocerids on M2, the models showed only weakly significant p-values with a model of CSR. We cannot reject the remaining models, as the number of points is likely too low. In contrast, the ammonoid pattern did not significantly deviate from CSR (Fig. 4c,d). This was especially true when we considered the gaps (M2x). Furthermore, an inhomogeneous Poisson model fitted very well to the data. While the DCLF test was not able to rule out a clustering model, the fitted model parameters showed that the best-fit model produced large-scale clusters that essentially encompassed the whole observation window. Thus, we conclude that there is no evidence for a similar pairwise clustering in the ammonoid distribution. For all three patterns on M2, choosing the gap-corrected or the uncorrected observation window had almost no influence on the summary functions, probably because only 9% of the area was missing and thus, only few additional points would be expected.

### Size

**Figure 6:**
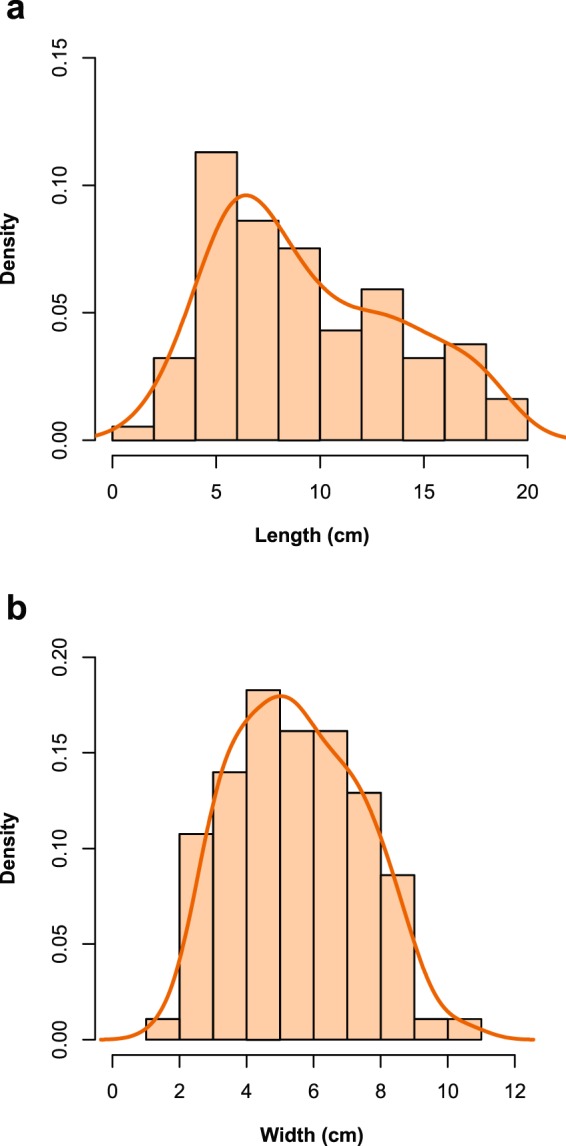
**S**ize distributions of the oncocerids on M1 and M2 as histograms and Kernel-smoothed density estimates. (**a**) length of the specimens and (**b**) widths of the specimens. Because of the conical shape of the specimens, a skewed distribution is to be expected in the length of the specimens. The width distribution is nearly symmetrical, thus showing no sign of a sexual size dimorphism. Note that the size of a specimen strongly depends on the position of the erosional surface.

As we recorded the shapes of the specimens, we could also measure the dimensions of the conchs. On average, the oncocerids on both surfaces had a length of 94 mm (median = 83 mm) and a mean width of 55 mm (median = 54 mm). As a comparison, orthocerids were significantly smaller, with a mean length of 55 mm (median = 46 mm) and a mean width of 16 mm (median = 15 mm). The size distributions of the oncocerids are shown in Fig. 6. Conch length was slightly skewed, while the width was almost symmetrically distributed (Shapiro-Wilk test for normality: p = 0.18). There is no strong indication in the data for distinct size classes. On the contrary, inspecting individual pairs of specimens shows that they are in many cases (but not always) of similar size. While it is conceivable that the size distribution is resulting from two similar normal distributions, testing for this is largely hampered by the fact that all specimens are only seen as two-dimensional sections at a presumably random point of the conch on the surface of the horizon. Thus, the distributions in Fig. 6 do not accurately reflect the true size distributions of the population and size tends to be underestimated. This bias is the reason why we did not explicitly test for a possible sexual dimorphism.

### Orientation

**Figure 7:**
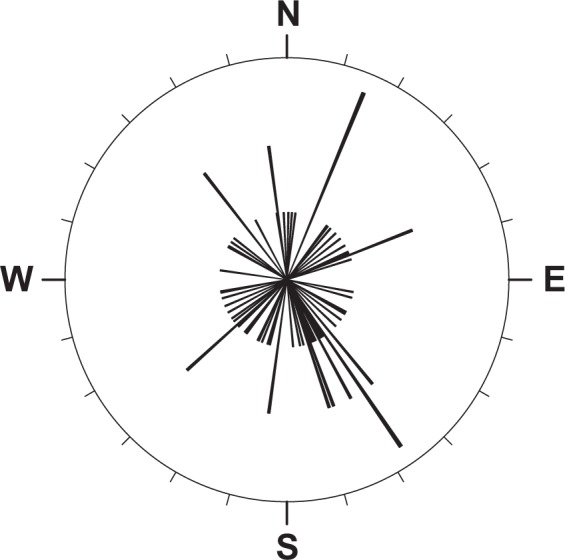
Rose diagram of the orientations of oncocerids on M1. Directions are taken in adoral direction. The shortest lines represent one specimen each.

Concerning the orientation of the specimens on M1, the angles had a mean resultant length of $\bar{{\rm{R}}}$ = 0.18 and a mean direction of $\bar{{\rm{\theta }}}$ = 301° (ESE) with an angular standard deviation of v = 106°. These values indicate a high dispersion of the angles, as is also shown in the rose diagram in Fig. 7. Furthermore, the Kuiper's test resulted in a weakly significant *p*-value ~ 0.09. As such, we cannot rule out a uniform distribution of the directions. In any case, the results indicate that the shells are either only weakly or not at all current-aligned.

## Discussion

The results falsify the null hypothesis of a random distribution of the oncocerids and support a clustering model. In contrast, the ammonoids do not show evidence for a similar clustering pattern on the same bedding plane. Although ammonoid and oncocerid conchs had different hydrodynamic properties and thus certainly behaved differently under the same current-regimes, we think that post-mortem transport was minimal (see below). This suggests that the cause for the clustering pattern is biological rather than taphonomic. However, we cannot rule out that the ammonoids ended up more randomly (i.e. because of post-mortem drift) in the environment due to differences in mode of life. Although some of the pairs seem to be aligned with each other (compare Fig. 1), we could not confirm this as a general trend.

### Abiotic causes and potential biases

Because cephalopods are motile animals and their conchs were nearly neutrally buoyant and thus prone to transport (see below), we have to be cautious when drawing conclusions from their spatial distribution on a bedding plane. We should ensure first that the point pattern at least approximately represents the positions where they died. There are also a few other factors that could possibly cause or bias the observed pattern:

#### Measuring inaccuracies

Because of the asperity of the terrain, flexibility of the plastic sheet (see methods) and slightly windy weather at the day of data collection, a slight measuring error in the point pattern is to be expected. However, as the distance between clusters is usually larger than the distance between individual specimens, the effect on the results is probably minor.

#### Exposed surface

We only corrected for gaps on M2 and therefore, there is probably a slight error in the models for M1. Nevertheless, the area of M1 is larger, the gaps are probably more or less randomly distributed and the effect of the gaps on the intensity is relatively low in M2; thus, we think that this influence is rather minimal. Moreover, because the Gauss-Poisson model explicitly involves randomly removing points from the pattern, we already incorporated this effect at least partially into the model to some extent.

#### Time averaging

It appears highly unlikely that the specimens are all of the exact same geological age. Nevertheless, the similar preservation, size, shape and orientation of many of the paired specimens (compare Fig. 1) makes it well conceivable that those died at the same time. However, it is impossible to say whether multiple pairs were deposited in a single season or if they represent different years that may have been separated by long timespans. Perhaps, recurrent storms (see Ebert^42^ for estimations of the depositional depth) removed or destroyed specimens and only prolonged periods with relatively calmer weather conditions kept the specimens intact and in place. Thus, it is possible that the ammonoids were deposited over a prolonged period of hundreds or thousands of years, while the deposition of the oncocerids was more instantaneous.

#### Catastrophic mass mortality

Among cephalopods, belemnites are especially well known for mass accumulations, resulting in so-called “belemnite battlefields”^43^. The processes behind these accumulations are varied, but catastrophic mass mortality is sometimes a possible cause^43^. Accumulations of this type can be identified by 1) the presence of the entire biota, 2) the presence of the whole age range of the taxon in question and 3) associated evidence for mass mortality such as volcanic ash layers, anoxia and algal blooms^43^. The Jurassic Peterborough Member in southern England is a notable example, because it also contains belemnite battlefields with high proportions of pairs^44^. These cases were interpreted as resulting from catastrophic mass mortality, rather than post-spawning mortality^44^. However, in contrast to our example, these deposits contain ample evidence for anoxia and in addition, the pairs comprise multiple species and represent a broad age range^44^. While we cannot exclude the presence of multiple oncocerid species in the “lower marker bed”, they are invariably adult. Additionally, although the entire biota is present as in condition 1), the high density of fossil specimens is not unusual for Devonian sections of the Tafilalt, where condensation is a typical phenomenon^45^. Thus, this factor alone is no indication for catastrophic mass mortality, especially as there is no supporting evidence for any catastrophe. Furthermore, catastrophic mass mortality alone can provide no explanation for the frequent pairing of specimens. In the Peterborough Member example, the pairs were likely caused by cannibalistic predation, combined with anoxia^44^. A similar mechanism appears unlikely for the oncocerids of the “lower marker bed”, since they were probably poor swimmers^22^ and thus not actively predatory. Furthermore, the cluster sizes would be expected to be larger than two and the shells would be more destroyed or show clearer signs of predation under these circumstances^43^. As explained below, it is also unlikely that they were interrupted by anoxia during the mating process.

#### Post-mortem transportation

Cephalopod shells may drift considerably after death, as evident in the extant *Nautilus* and *Spirula* although recent studies show that extended periods of drift are probably more the exception than the rule^46^. This point might be less relevant in our case, as already short-distance transport could confound the results. Therefore, we consider it crucial to assess this point. In contrast to *Nautilus*, oncocerids were considered as posthumous sinkers^20^. This also fits with the observation that breviconic cephalopods are commonly restricted to shallow depositional settings^22^. Mathematical models and documentations of broken septa (caused by implosion?) suggest that breviconic shells sank either directly after death or after a short period of drifting^47^. In any case, it is difficult to interpret the post mortem fate of oncocerids because of the lack of modern analogues. Nevertheless, it appears likely that the shells sank soon after their demise because the shell was –for energy efficiency reasons– probably slightly negatively buoyant, similar to modern day *Nautilus*^48^. The next step is less trivial to reconstruct. In most studies, it is assumed that cephalopod shells would become positively buoyant after the decay of the soft parts because the soft parts were slightly denser than seawater^47^. However, the siphuncle is relatively large in oncocerids and thus less easily sealed off by sediment or other particles than in cephalopods with narrow siphuncles (as for example in *Nautilus*). Therefore, when the soft parts had fallen out of the body chamber, water could easily enter the siphuncle and at least slightly increase the density of the conch. But more importantly, because the oncocerid connecting ring is highly porous with a larger relative surface than in *Nautilus*^49^ and the osmotic pumping capacity is gone together with the siphuncular epithelium^50^, water would soon be able to flood the chambers at least partially. In addition, gas would be probably less easily trapped within the siphuncle because of the straight conch shape. Another important question is also if and how long the apex remained intact, because breakage of this fragile shell part would have accelerated water logging of the phragmocone. By comparison, the apex of *Nautilus* and other coiled forms is better protected by the overlying whorls.

In the material reported here, most specimens retained at least some intact septa; this suggests that the specimens were deposited at a depth close to where they died, because a marked change in ambient pressure would have caused the destruction of the septa. Phragmocone implosion is also unlikely because the Tafilalt Platform was most likely above the implosion depth of an oncocerid phragmocone^42,47^. Lastly, there are indications that oncocerids were more efficient than *Nautilus* in using their siphuncle for buoyancy control^49^. Thus, if the animals died near the sea floor (for example because of reproduction), their chambers were potentially already filled with water to a certain degree when they died.

Even if drift appears to be unlikely, post-mortem transport can also occur along the sea floor. Different types of shell breakage patterns indicative of transportation in *Nautilus* have been shown by Wani^51^ and allowed us to carry out cursory assessments, whether significant post-mortem transport took place in our sample. However, none of our specimens showed breakage patterns similar to these and most damages are likely rather caused by diagenesis or erosion.

Despite post-mortem transport being unlikely in the oncocerids, we are unable to rule out the same for the ammonoids that had different hydrostatic properties and are perhaps more prone to post-mortem transport. Thus, it is possible that the random distribution of the ammonoids is in fact resulting from post-mortem drift.

In summary, together with the non-current alignment of the specimens, we found no indications that extensive post-mortem transportation took place in the oncocerids. We also remark that post-mortem transport cannot explain the pairwise clustering pattern but may be responsible for the random distribution of the ammonoids.

### Speculations on the reproductive strategy of oncocerids

The close association of pairs of the same species is rare in the fossil record, but is sometimes interpreted as death occurring during mating. Examples are Eocene turtles from the famous South German Messel Pit^52^ and one case each of Carboniferous ammonoids from the Bear Gulch Limestone of Montana and of Jurassic ammonites from the Plattenkalke of Eichstätt in South Germany^53^. For cases like these, the term “distraction sinking” has been coined^53^. Under these conditions, anoxic or poisonous bottom waters presumably ended the mating process and killed the couples. Another glimpse into the reproductive behaviour of fossil organisms can be seen in the clustering of different size classes of Ediacaran organisms at Mistaken Point, Newfoundland, which was analysed using similar techniques as in this study^54^.

In contrast to the former two examples, the oncocerids in the “lower marker bed” at Hamar Laghdad are not in direct contact with each other. In addition, they are not embedded in sediments with properties suggesting anoxia or poisonous environments. Independent of the cause of their demise, our analyses and the fact that all specimens on the surface appear to be adult because of their contracted apertures support the assumption that the specimens represent reproductive pairs. Because of the high density of pairs, a sudden death during mating appears unlikely, even more so when considering the lack of evidence for any process that could have killed the animals. The water depth was probably low (Ebert^42^ estimated it around 50 m; see also Wendt^55^ and Töneböhn^56^) and thus, the sea floor was well-oxygenated (also supported by the abundance of trace fossils) in contrast to the other two examples^42^. In our opinion, it is more likely that the animals died shortly after copulation and spawning, which would imply that these oncocerids were semelparous. In this regard, they would be more similar to many modern coleoids than to nautilids (which are iteroparous). However, one important difference is that in semelparous coleoids, death does not occur immediately after mating, since the female first lays her eggs, which can take a couple of days^57^. In this scenario, male and female do not die at the same spot. Moreover, many coleoids such as *Loligo* are known to accumulate for mass spawning events, which would result in such an abundance of specimens, that it would completely overshadow a pairwise clustering pattern. Considering the probably slow sedimentation rates of the “lower marker bed”^42^, the preservation of pairs was probably a rather rare event.

Although sexual dimorphism has been suggested for oncocerids, we did not find any indications for distinct size classes in our sample. If sexual dimorphism is present, it is not immediately obvious. The *in situ* preservation of the specimens makes comparison difficult. Furthermore, the size of the specimens strongly depends on the surface of erosion, where the specimens have been cut. This factor would likely overshadow any clear relationship. However, the size distribution of the conch width is close to a normal distribution, thus it is conceivable, that the specimens belong to the same species, although there is no guarantee for this. The skewed distribution of the length of the specimens can be explained by the preservation of their conical, rapidly expanding conch shape (compare *Pachtoceras*? *laghdadense*^31^), i.e. randomly cutting them in the longitudinal axis or slightly oblique would result in a higher concentration of seemingly short shells. It may be expected that the pairs would be preserved with the apertures close to each other. However, assuming copulation took place in the water column (perhaps close to the sea floor) as in recent cephalopods, their orientation as a fossil would largely depend on how they would eventually sink to the bottom and if any post-mortem disturbance would occur.

From the above it appears plausible that at least the oncocerids encountered at Hamar Laghdad considerably differed in their reproductive strategy from both coleoids and nautilids. Assuming that both parents died while they were still in close proximity, we propose two alternative hypotheses on their reproductive strategy, which cannot be fully tested at this point as we have no evidence of the soft-part anatomy. They serve as examples to show how different reproductive strategies could result in a pattern such as the one seen at Hamar Laghdad.

#### External fertilisation

After the female had laid her eggs, they were immediately fertilised externally by the male. Shortly afterwards both parents would die of exhaustion. This reproductive strategy would somewhat resemble some extant teleost species such as salmon^58^. Although crown-group cephalopods are all internal fertilizers, there is no evidence that this was also the case in early cephalopods. Molluscan phylogeny is still debated^59,60^, but many other taxa including the likely closest relatives Monoplacophora are external fertilizers^61^. Thus, it is conceivable that internal fertilisation in cephalopods did not evolve at the base of the group and is a derived habit that evolved in cephalopods closer to the crown group. The possible late appearance of internal fertilisation is supported by the fact that recent cephalopods are not internal fertilisers in the strict sense, as in most species the female stores the spermatophores and the eggs are only fertilised upon spawning^57^. Many recent coleoids possess either a hectocotylus or terminal organ (penis) to transfer spermatophores to the female, while the spadix fulfils the same role in *Nautilus*^57^. The complexity and wide variability of the spermatophore transfer and storage mechanisms within coleoid cephalopods compared to the relative simplicity of the strategy employed by *Nautilus*^62^ may be seen as further indication for this hypothesis. This scenario of course depends on whether oncocerids belong to the crown group or not (see discussion below).

#### Shared parental care

Fertilisation was internal, but the period between fertilisation and egg laying was perhaps rather short. To increase survival chances, the male guarded the female at least until the eggs were laid, possibly both parents even protected the clutch. Perhaps, this was combined with brooding, possibly even within the body chamber (ovoviviparity) as suggested by Mutvei^26^. A similar behaviour of limited parental care is known from females of several extant octopod species^2,63^.

Note that neither the recent *Nautilus* strategy nor any of the coleoid strategies would result in a pattern of pairwise preservation. Although the analyses provide statistical support that the oncocerids were clustering in pairs at the time of their death, the cause of the clustering (i.e. semelparity) is hard to pin down with certainty and thus remains speculative. It is therefore also possible that the pattern is not directly related to reproduction but to other behaviours such as rivalling male fighting^53^, predation^44^ etc. Nevertheless, taking all the evidence together, we think that a semelparous behaviour provides the best explanation but requires further supporting evidence. We also want to highlight that the analyses do neither confirm nor support semelparity or iteroparity for the ammonoids, as their random distribution pattern could also result from other processes such as post-mortem drift due to different hydrostatic properties of the shell or modes of life – regardless of their reproductive strategy.

### Evolution of semelparity in cephalopods

**Figure 8:**
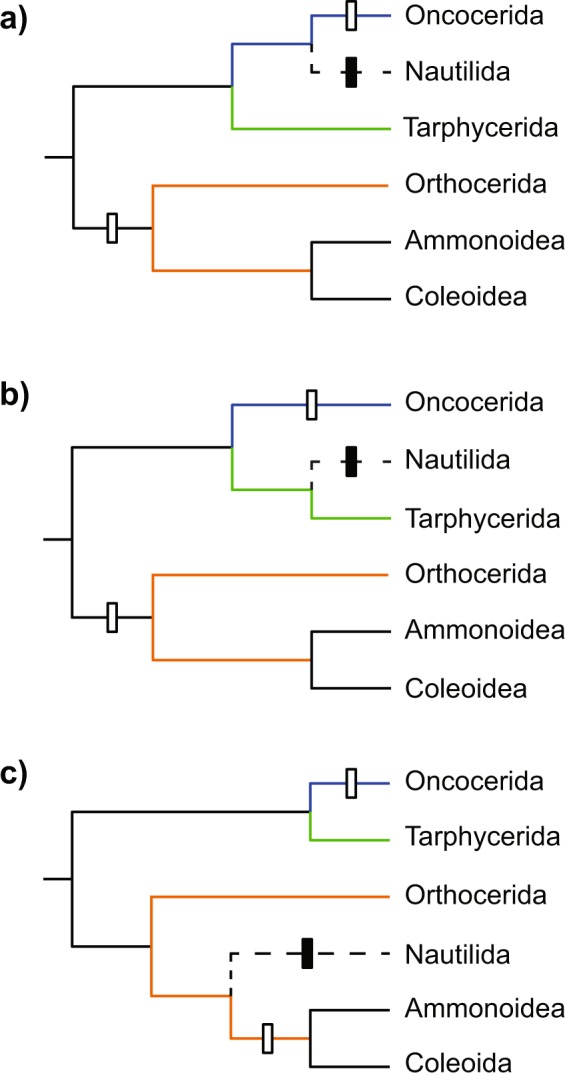
Evolution of semelparity within cephalopods according to different phylogenetic hypotheses regarding the origin of the Nautilida: (**a**) from Oncocerida^65^, (**b**) from Tarphycerida/ Barrandeocerida^24^ and (**c**) from Orthocerida^66^. White rectangles represent acquisition of semelparity assuming that iteroparity is the primitive condition. Black rectangles represent an alternative scenario where semelparity is primitive and lost in the Nautilida, which evolved iteroparity instead. Possibly paraphyletic groups are shown in colour: Oncocerida (blue), Tarphycerida (green) and Orthocerida (orange). Fossil groups are assumed to be similar in reproductive strategies to their closest relatives. In all three cases with primitive iteroparity, it is possible that the transition to semelparity occurred after the origin of the Orthocerida and both strategies existed within this group.

Recent cephalopods exhibit a wide range of reproductive strategies^2^. The strategies of extinct taxa are for the most part unknown, especially in those taxa that are only distantly related to modern forms, as is the case with the Oncocerida. The evolutionary relationships among early Palaeozoic cephalopods are still poorly understood and the published phylogenetic hypotheses quite different^24^. Nevertheless, it appears likely that the Oncocerida is the sister group of the Tarphycerida, since both can be derived from the Early Ordovician Bassleroceratidae^64^. Furthermore, in contrast to the long-standing classic hypothesis of Flower & Kummel^65^, the origin of the modern Nautilida appears to lie not within the Oncocerida but within another lineage^24,66^. If the nautilids evolved from the Orthocerida (which in turn gave rise to the Bactritida that are considered ancestral to coleoids and ammonoids), oncocerids would have diverged from crown-group cephalopods already during the Ordovician^66^. In this case, statements about their soft part anatomy or other biological aspects cannot reliably be based on comparisons with modern cephalopods. In contrast, if the Nautilida descended directly from the Oncocerida or Tarphycerida, the oncocerids would belong to the cephalopod crown group and characters shared by recent coleoids and nautilids can be assumed to be present in primitive oncocerids as well (extant phylogenetic bracket^67^). Unfortunately, in terms of reproduction, this comes with the pitfall that both recent groups have such widely differing reproductive strategies. Under the assumption that the *Nautilus* strategy is primitive in cephalopods, semelparity would have appeared at least twice independently in cephalopods, regardless of the phylogenetic hypothesis (Fig. 8). The alternative scenario would be that semelparity is primitive and iteroparity evolved just once somewhere along the *Nautilus* branch.

While semelparity and iteroparity are often viewed as discrete life history traits, recent studies have shown that parity is better described as a continuum with both strategies as extremes of the spectrum^68^. This is furthermore exemplified by the wide range of reproductive strategies within the coleoid clade^2^. The duration over which females lay eggs and the seasonality, if any, is not well known in *Nautilus*^1^, suggesting that the two reproductive endpoints of cephalopods may not be as distinct as previously suggested. Therefore, the transition from iteroparity to semelparity or vice versa was likely not a single distinct event during cephalopod evolution.

However, as the knowledge on the reproduction of fossil cephalopods is so patchy, none of these scenarios can be favoured at the moment. Accordingly, more studies both on reproduction and phylogeny are needed. Rather importantly, this study shows that reproductive strategies of fossil cephalopods are more diverse than anticipated and consequently, only more direct or indirect evidence for reproduction can elucidate the true range of this variation. Lastly, we provided a method to test for semelparity in fossil cephalopods or other organisms more directly, if circumstances are carefully taken into account.

## Methods

### Data collection

The surface of the study area was covered by a 4 × 5 m transparent plastic cover sheet. We then searched for oncocerids below the plastic sheet. As orthocerids also occur in the same layer and the shape of the cephalopods varies depending on how much and in which angle the specimen was eroded, we used close septal spacing, a contracted aperture and where visible the broadly expanded siphuncle as criteria to unequivocally identify oncocerids. Generally, oncocerids are significantly larger and have a much greater diameter than orthocerids on the study surface (see results). We traced the outline and the last septum of the specimens on the sheet with a waterproof marker. After tracing all specimens below the sheet, its position was marked on the rock surface and the plastic sheet was moved to the side, adjacent to the previous position. The specimens below the sheet were then marked as described previously, but with a different colour. The orientation and position of the previous sample was also noted in order to reconstruct the whole surface later. This procedure was performed in four different sheet positions, covering in total a continuous area of 80 m^2^. This surface is referenced here as M1.

In order to compare the data obtained from the oncocerids with other cephalopods, we repeated the same procedure on roughly the same bedding plane, but this time we traced all available cephalopods, i.e. ammonoids, orthocerids and oncocerids. The surface area of the second sheet was 2 × 6 m, which was laid out two times, resulting in 24 m^2^. In some places, the top of the horizon was covered by sand, calcrete or cracks, making it impossible to detect cephalopods and causing gaps in the recorded pattern. In order to test the influence of these gaps, we recorded them as well on the second sheet. We refer to the second surface as M2 and as M2x for the surface including gaps.

To digitise the collected data, we cut the plastic sheets into numbered pieces of 1 × 1 m, which were then photographed at a standardised camera angle and distance. Subsequently, we digitally reassembled the pictures and traced the outline of the specimens and gaps. The coordinates of each specimen – taken at the centre of the outline – were compiled into a data set. Data exploration and analyses were performed in R version 3.4.4^69^. For circular statistics, we used the CircStat package^70,71^ and for spatial point pattern analyses the spatstat package^72,73^.

### Post-mortem transportation

Before analysing the spatial distribution of the specimens, we first considered first-order effects, such as post-mortem transportation. This was at first done qualitatively by comparing the preservation of the specimens to the existing literature on post-mortem transportation of cephalopod shells^46,51,74^.

As another, more quantitative test for transport, the possibility of palaeo-currents was considered. In this regard, the orientation of cephalopod and other invertebrate shells has often been used as a tool to reconstruct the depositional settings^75–77^. We deduced the shell directions from the recorded shapes of the specimens and calculated the mean resultant length $\bar{{\rm{R}}}$ (dispersion), the mean direction $\bar{{\rm{\theta }}}$ and the angular standard deviation v of the angular data^78^. To test for uniform distribution of the shell directions we used the modified Kuiper's test of Mardia & Jupp^78^.

### Spatial point pattern analysis

The distribution pattern was analysed using spatial point pattern statistics and modelling approaches^73^. A point pattern consists of a set of coordinates lying within an observation window. A point pattern is a realisation of a point pattern process. An entirely random point pattern exhibits complete spatial randomness (CSR), which can be obtained by modelling a homogenous Poisson process (see below).

To transform our data to a point pattern, we assigned coordinates approximately to the centre of each specimen. While this has the potential to introduce systematic errors, we consider them as negligible because inaccuracies in the field caused by wind, uneven terrain or the difficulties in aligning the cover sheet likely have a larger effect. Additionally, the shape of the specimens on the bedding plane might not be an accurate representation of the fossils, as it strongly depends on the surface of erosion.

For the point patterns, we calculated the following values and statstics:

#### Mean intensity

$\bar{\lambda }$ describes the average number of specimens per area. If the intensity is constant, it is equal to the average density (specimens per square meter). The intensity can also be spatially varying; in that case λ is a function of the Cartesian coordinates, a numerical or a categorical covariate^73^. A point pattern process is called stationary when the intensity is constant (homogenous) and non-stationary when the intensity is variable within the observation window (inhomogeneous). The intensity distribution was evaluated by generating kernel-smoothed intensity maps of all observation windows. This allowed us to evaluate whether the intensity distribution was homogenous or inhomogeneous.

#### Ripley's K

The summary statistic *K(r)* was introduced by Ripley^79^ and has been applied in numerous ecological studies (see Wiegand & Moloney^80^ and references therein). It describes the average number of points that lie within a circle with radius *r* divided by $\bar{\lambda }$; the latter action is performed in order to standardise the data and make it comparable. To correct for sampling biases due to the shape of the observation window, we used the translation edge correction as implemented in spatstat^72^. We used the same edge correction in all analyses. If the empirical K-function $\hat{K}$ *(r)* lies significantly below the theoretical *K*_*Poisson*_*(r)* at a certain value of *r*, this indicates that there are fewer points within this distance than would be expected than under the current model (segregation) and in the opposite case that there are more points than expected than under the null model (aggregation).

#### L-statistic

*L(r)* is essentially the same as Ripley's K, but transformed into √K in order to visualise the null model of a homogenous Poisson process (CSR) as a straight line^73^ and make it easier to interpret.

#### Pair correlation function

In contrast to Ripley's K and the L-statistic, the pair correlation function *g(r)* only takes into account points that lie within the same distance^73^. A value of *g(r)* = 1 indicates no correlation (complete spatial randomness), while values *g(r)* < 1 indicates segregation and *g(r)* > 1 aggregation. To correct for bias at small distances, we used the modification implemented in spatstat (divisor = d)^73^.

#### Edge correction

To correct for bias due to unobservable points outside the observation window, we applied the translation edge correction for all summary functions^73^. A comparison between different methods of edge correction showed that they produced practically identical summary functions. This follows the recommendation of Baddeley *et al*.^73^ in that it is of minor importance, which method is chosen, as long as any edge correction is applied.

#### Monte-Carlo simulations and significance tests

In the next step, the data were fitted to different point process models by finding optimal parameters of the models. Details of the models and fitting techniques are given below. The fitted models were then used for Monte-Carlo simulations of point patterns. For every model, 99 simulations were performed and corresponding summary functions and envelopes with minimum and maximum values were calculated and plotted. The Diggle-Cressie-Loosemore-Ford (DCLF) test was applied to infer the statistical significance^73^. As discussed by Baddeley *et al*.^81^, significance testing for spatial models can be problematic and a non-significant p-value does not necessarily confirm a model (though significant p-values still reject a model). Additionally, the same point pattern can arise from a variety of point pattern processes^73^. Thus, caution is generally advised when formulating models for point pattern processes. We therefore only formulate models that represent a real possibility for the formation of the point patterns.

#### Homogeneous poisson model

The most basic model of a point process is complete spatial randomness (CSR), which assumes that the points are randomly distributed over a given area with a constant intensity ($\bar{\lambda }$). Thus, $\bar{\lambda }$ was used as obtained from the data to fit the model. Although this model is mostly unrealistic in ecological processes, it is still valuable as a null hypothesis that requires hardly any assumptions.

#### Inhomogeneous poisson model

In case that the homogeneous Poisson models cannot explain the data, one may apply an inhomogeneous Poisson model^73^. As in the previous model, the points are randomly distributed, but the intensity varies over the study area according to a density function. As our dataset lacks useful covariates, we used the Cartesian coordinates. In some studies, the intensity function of the data itself is used as intensity background^54,80^. However, it runs the risk of overfitting the model and we did not apply it here. The fitted intensity function in our case thus may represent a gradient of an unknown variable, such as (palaeo-) terrain, outcrop irregularities, palaeocurrents or other palaeoecological conditions.

#### Thomas cluster model

If the previous models can be excluded, the point pattern may be a realisation of a clustering process. Here, we used a Thomas cluster model^73,82^. Under this model, which is a case of a Neyman-Scott process^73^, a homogeneous Poisson point pattern with parent intensity κ is first produced and the resulting parent points are then replaced by clusters with a Poisson distributed cluster size σ and a cluster radius *r*. The model was fitted to the data using the Minimum Contrast method^83^, which fits the theoretical K-function of the model to the empirical K-function of the observed pattern. For optimisation, we used the Nelder-Mead algorithm, which is the standard in spatstat^73^.

#### Gauss-poisson model

The Thomas cluster model (or any of the standard cluster models) assumes that the cluster size is Poisson distributed. However, we suspect here that the specimens preferentially cluster in pairs. Consequently, we fixed the cluster size at σ = 2.0 and added a random modifier that removed one of the two points with probability 1-*P*. This corresponds to the Gauss-Poisson model^84,85^ but with the modification that the distance between the points is random and not fixed in our case, i.e. the points are uniformly distributed within a disc with radius *r*. As in the Thomas cluster model, parent points are randomly distributed in the observation window with intensity κ. Fitting the Gauss-Poisson model to the data was more challenging than previous models, because the theoretical summary function of the modified Gauss-Poisson model is unknown. Thus, we estimated the theoretical K-function of the model by simulating 100 patterns of the model each iteration of the model fitting process. As for the Thomas cluster model, the fitting process involved the Minimum Contrast method (see above).

## Supplementary information

Supplementary information.

## Data Availability

All data generated or analysed during this study are included in this published article (and its supplementary information files).
